# Incidence and virulence characteristics of *Aeromonas* spp. in fish

**DOI:** 10.14202/vetworld.2017.34-37

**Published:** 2017-01-12

**Authors:** Ashraf M. Abd-El-Malek

**Affiliations:** Department of Food Hygiene (Meat Hygiene), Faculty of Veterinary Medicine, Assiut University, 71515 Assiut, Egypt

**Keywords:** *Aeromonas hydrophila*, fish, hemolysin, lipase, protease enzyme, public health

## Abstract

**Aim:**

This study was conducted to evaluate the presence of *Aeromonas* spp. in raw and ready-to-eat (RTE) fish commonly consumed in Assiut city, Egypt, and to determine virulence factors due to they play a key role in their pathogenicity.

**Materials and Methods:**

A total of 125 samples of raw and RTE fish samples were taken from different fish markets and fish restaurants in Assiut Governorate and screened for the presence of *Aeromonas* spp. by enrichment on tryptic soy broth then incubated at 30°C for 24 h. Plating unto the sterile Petri dishes containing *Aeromonas* agar base to which *Aeromonas* selective supplement was added. The plates were incubated at 37°C for 24 h. Presumptive *Aeromonas* colonies were biochemically confirmed and analyzed for pathogenicity by hemolysin production, protease, and lipase detection.

**Results:**

The results indicated that raw fish were contaminated with *Aeromonas* spp. (40% in wild and 36% in cultured Nile tilapia). Regarding RTE, *Aeromonas* spp. could be isolated with the percentage of 16%, 28% and 20% in fried Bolti, grilled Bolti and fried Bayad, respectively. Out of 35 isolates obtained, 22 were categorized as *Aeromonas hydrophila*, 12 were classified as *Aeromonas sobria* and *Aeromonas caviae* were found in only one isolate. The virulence factors of *Aeromonas* spp. were detected and the results showed that all isolates produced of hemolysin (91.4%), protease (77.1%), and lipase enzyme (17.1%).

**Conclusion:**

This study indicates that the presence of *A. hydrophila* with virulence potential in fresh and RTE fish may be a major threat to public health.

## Introduction

The most popular and commonly consumed freshwater fish species in Egypt are Nile tilapia (Bolti) and Bayad (*Bagrus bayad*). Grilled fish is food for common consumption in different parts of the world as well as in Egypt. Furthermore, frying of fish is practiced in some parts of the world.

*Aeromonas* is an emerging pathogen and is recognized to cause a variety of diseases in humans. This pathogen is associated with food poisoning and some human diseases as gastroenteritis and extraintestinal symptoms such as soft-tissue, muscle infections, septicemia, and skin diseases in humans [1].

Of the *Aeromonas* spp., *Aeromonas hydrophila*, *Aeromonas* *Sobria*, and *Aeromonas* *caviae* have been incriminated as the main causes of *Aeromonas* associated human diseases [2].

It has been established that hemolysin is a virulence factor contributing to the pathogenesis of *A. hydrophila* infection. Furthermore, fish plays an important role in the transmission of *Aeromonas* spp. to humans [3].

There is scarce of the published information and limited studies about the prevalence of *Aeromonas* spp. especially *A. hydrophila* and their virulence in raw and ready-to-eat (RTE) fish in Assiut city, Egypt.

Keeping in view the importance of these pathogens, therefore, the aim of this study was conducted to isolate and identify of *Aeromonas* spp. from raw fish and RTE fish samples in Assiut city, Egypt. In addition to determine the most virulence factors (such as hemolysin, protease and lipase) which play a key role in their pathogenicity.

## Materials and Methods

### Ethical approval

Not required for this study.

### Sampling and isolation of *Aeromonas* spp.

Raw fish samples consisted of wild Nile tilapia and cultured Nile tilapia (25 of each) which was randomly collected under aseptic conditions from different fish markets at Assiut city. Raw fish samples were stored in icebox with an appropriate quantity of crushed iced for transportation to the laboratory within 1 h. Regarding RTE fish samples included grilled Bolti and fried Bolti as well as fried Bayad samples (25 of each) which were collected as sold to the consumers in sterile plastic bags from different fish restaurants in Assiut city. All samples were labeled and transferred directly within 1 h to the laboratory, where immediately examined. Preparation of samples performed as recommended by Sanaa [4]. Isolation was done by enrichment on tryptic soy broth (Biolife, CP4712) then incubated at 30°C for 24 h. Plating unto the sterile Petri dishes containing *Aeromonas* agar base (Biolife, CN0801) to which *Aeromonas* selective supplement (Ampicillin) was added. The plates were incubated at 37°C for 24 h [5]. Presumptive *Aeromonas* colonies were biochemically confirmed according to Table-1 [6].

**Table-1:** Biochemical characteristics of different *Aeromonas* spp. [6].

Biochemical tests	*A. hydrophila*	*A. caviae*	*A. sobria*
Esculin hydrolysis	+	+	-
Gas from glucose	+	-	+
VP	+	-	V
Indole	+		
Citrate	+	+	+
Larabinose	+	+	-
H_2_S production	+	-	+
Hemolysis	+	V	V

+=Positive, −=Negative, V=Variable (50%), VP=Voges Proskauer

### Detection of some virulence factors of *A. hydrophila* [7]

#### Hemolysin production

*A. hydrophila* tested for hemolysin production on blood base agar (Britania, Argentina) supplemented with 5% sheep blood. A loopful of an overnight growth from nutrient agar was cultured on blood agar by streaking method, incubated at 37°C for 24 h.

#### Production of protease

Protease was determined on 2% agar-agar (Qualikems, India) containing 10% (w/v) skimmed milk.

#### Lipase detection

Lipase detection was performed on olive oil with phenol red agar. The serial diluted bacterial samples were plated on phenol red agar and incubated at 37°C overnight. The phenol red agar plates were prepared by incorporating phenol red (0.01% w/v), olive oil (0.1% v/v), CaCl_2_ (0.1% w/v), and agar (2% w/v). The change in color of phenol red was used as an indicator for lipase activity, where lipase producing bacteria will turn the dye into yellow color.

## Results and Discussion

### Incidence of *Aeromonas* spp. in raw fish samples

The obtained data in Table-2 revealed that *Aeromonas* spp. could be isolated from wild and cultured Nile tilapia samples with the percentage of 40% and 36%, respectively. *A. hydrophila* strains could be isolated from wild and cultured Nile tilapia samples with the percentage of 16% and 12%, respectively.

**Table-2:** Incidence of *Aeromonas* spp. in examined raw fish samples.

Samples	No.	N (%)			

		*Aeromonas* spp.	*A. hydrophila*	*A. caviae*	*A. sobria*
Wild Nile tilapia	25	10 (40)	4 (16)	1 (4)	5 (20)
Cultured Nile tilapia	25	9 (36)	3 (12)	0 (0)	6 (24)
Total	50	19 (38)	7 (14)	1 (2)	11 (22)

Regarding *A. sobria*, it could be isolated only from wild and cultured Nile tilapia in incidence of 20% and 24%, respectively. Meanwhile, only wild Nile tilapia contaminated with one isolate of *A. caviae* with the percentage of 4% (Table-2).

The incidence of *Aeromonas* spp. was higher (40%) in raw fish markets than that in aquaculture (36%) (Table-2) which may be attributed to post-harvest contamination during handling, transportation, and selling through fishermen and fish vendors [8]. In comparison with the obtained results outlined in Table-2, nearly similar results (39.58%) recorded by Gupta *et al*. [9] who found *Aeromonas* spp. in 38 samples of raw fish. On the contrary, higher results (100%) of *Aeromonas* spp. in tilapia reported by Manna *et al*. [10] in a related study in India. On the other hand, lower incidence (12% and 34%) of *Aeromonas* spp. obtained by Elshahid *et al*. [11] and Alhazmi [12], respectively, from raw fish samples. In this study, 3 *A. hydrophila* strains (with a percentage of 12%) were isolated from 25 cultured Nile tilapia. On contrast, higher results achieved by El Deen *et al*. [13] who found that a total of 10 *A. hydrophila* strains (with a percentage of 25%) were isolated from 40 cultured Nile tilapia collected randomly from the ponds of private fish farm in Kafr El Sheikh Governorate, Egypt.

### Occurrence of *Aeromonas* spp. in RTE fish samples

*Aeromonas* spp. could be isolated from fried and grilled Bolti as well as fried Bayad samples with the percentage of 16%, 28% and 20%, respectively (Table-3). *A. hydrophila* strains could be detected in fried Bolti and grilled Bolti, as well as fried Bayad samples with the percentage of 12%, 24% and 20%, respectively (Table-3). Concerning *A. sobria*, it could be isolated only from grilled Bolti in incidence of 4% (Table-3).

**Table-3:** Incidence of *Aeromonas* spp. in examined RTE fish samples.

Samples	No.	N (%)			

		*Aeromonas* spp.	*A. hydrophila*	*A. caviae*	*A. sobria*
Fried Bolti	25	4 (16)	4 (12)	0 (0)	0 (0)
Grilled Bolti	25	7 (28)	6 (24)	0 (0)	1 (4)
Fried Bayad	25	5 (20)	5 (20)	0 (0)	0 (0)
Total	75	16 (21.3)	15 (20)	0 (0)	1 (1.3)

In comparison with the obtained results outlined in Table-3, lower percentage (2.27%) of *Aeromonas* spp. was isolated [9]. Meanwhile, a higher result (77.3%) of RTE fry fish was found contaminated with *Aeromonas* spp. [10].

The obtained data demonstrated that out of 35 *Aeromonas* spp. isolates obtained, 22 were categorized as *A. hydrophila*, 12 were classified as *A. sobria* and *A. caviae* were found in only one isolate (Tables-2 and 3). Consequently, *A. hydrophila* and *A. sobria* predominate among potentially pathogenic *Aeromonas* isolates from examined raw and RTE fish. Similarly, many investigators pointed out that *A. hydrophila* was the most common isolate from foods of animal origin [14].

Many researchers could isolate *A. hydrophila* from *Bagrus bayad* such as Hussien and Salman [15] who reported that the bacterial isolates from the fresh *B. bayad* samples were 9% *A. hydrophila* and Elshahid *et al*. [11] who could isolate *A. hydrophila* from freshwater *B. bayad* with percentage of 16%. The high contamination rate of RTE fish suggests recontamination after cooking caused by lack of hygiene and post-process contaminants from uncooked produce or contaminated water. The presence of *Aeromonas* spp. in grilled fish with large number (28%) than fried fish (16%) may be due to rapid grilling have been proved to be insufficient to kill all harmful microorganisms which may be present in raw fish prior to preparation. Further, the consumption of retailed grilled fish regarded as potential public health hazard.

### Detection of virulence factors of *A. hydrophila*

The results presented in Table-4 shown that hemolysin was produced by 86.4% of *A. hydrophila*, 100% of *A. sobria*, and 100% of *A. caviae*. In this study, the obtained results showed that *A. hydrophila* were able to hydrolyze the protein by protease enzyme (77.1%) when tested on skim milk agar. Furthermore, *Aeromonas* isolates in this study had the ability to hydrolyze fats by lipase enzyme in 17.14% when cultured on phenol red olive oil agar for 3-5 days at 37°C (Table-4).

**Table-4:** Prevalence of virulence factors tested from *Aeromonas* spp.

Species	Number of samples tested	Hemolysin activity		Protease activity		Lipase activity	
		
		+ve	%	+ve	%	+ve	%
*A. hydrophila*	22	19	86.4	14	63.6	5	22.7
*A. sobria*	12	12	100	12	100	1	100
*A. caviae*	1	1	100	1	100	0	0
Total	35	32	91.4	27	77.1	6	17.1

+ve=Number of positive strains, %=Percentage of positive strains

Comparing with the results illustrated in Table-4, *A. hydrophila* showed positive result for hemolysin production (100%), type beta (β -hemolysin), when cultured on blood agar medium [7]. There was a strong correlation between the hemolysin and the virulence of *A. hydrophila* isolates. In a study conducted by Hatha *et al*. [16] recorded that 100% of *A. hydrophila*, 50% of *A. sobria* and 77.8% of *A. caviae* exhibited hemolytic activity. Moreover, Erdem *et al*. [17] reported that *A. hydrophila* and *A. veronii* biovar sobria strains were found to possess strong hemolytic activity, whereas *A. caviae* strains were nonhemolytic. In this study, the achieved results showed that *A. hydrophila* were able to hydrolyze the protein by protease enzyme (77.1%) when tested on skim milk agar. On the other hand, higher percentage (100%) was recorded by other authors as Al-Fatlawy and Al-Hadrawy [7], Pandey *et al*. [18] who emphasized that *A. hydrophila* was producing protease enzyme which able to hydrolyze the protein when tested on skim milk agar. Another study recorded proteolytic activity in 94.8% of *Aeromonas* strains [17]. Furthermore, lipase enzyme was present in 17.14% of *Aeromonas* isolates (Table-4). Higher incidence recorded by Al-Fatlawy and Al-Hadrawy [7] who emphasized the ability of *A. hydrophila* to hydrolyze fats by lipase.

Preventive method should be taken during food preparation; fish should be thoroughly cooked before consumption and good personal hygiene and proper sanitation procedure should always be used to prevent human exposure to this disease [5].

## Conclusions

This study indicates that the presence of *A. hydrophila* with virulence potential in fresh and RTE fish may be a major threat to public health. Consequently, the public should be enlightened on the inherent danger that may accompany handling fresh fish or consumption of improperly cooked (either grilled or fried) fish.

## Authors’ Contributions

Study design, samples collection, laboratory work, and the manuscript writing were done by AMA. AMA has read and approved the final manuscript.
